# Supplementary material for: Does Extraesophageal Reflux Support the Development of Lung Adenocarcinoma? Analysis of Pepsin in Bronchoalveolar Lavage in Non-Smoker Patients
Source: Cancers (Basel). 2024 Jul 28;16(15):2687. doi: 10.3390/cancers16152687 (PMC11312250; doi:10.3390/cancers16152687)
Supplement: Supplementary file 1 [file cancers-16-02687-s001.zip › cancers-3086845-supplementary.pdf]

**Supplementary Table S1:** Characteristics of participants in the lung adenocarcinoma group.

| | | |
|---|---|---|
| **Peripheral lung adenocarcinoma** | 19 / 30 (63.33%) | |
| **Central lung adenocarcinoma** | 11 / 30 (36.67%) | |
| **TNM 8th edition** | IA2 | 2/30 (6.67%) |
| | IA3 | 4/3013.33%) |
| | IB | 2/30 (6.67%) |
| | IIA | 1/30 (3.33%) |
| | IIB | 2/30 (6.67%) |
| | IIIA | 6/30 (20.00%) |
| | IIIB | 3/30 (10.00 %) |
| | IIIC | 1/30 (3.33%) |
| | IVA | 1/30 (3.33%) |
| | IVB | 8/30 (26.67%) |
| **Mutation status** | | |
| ALK | 1/30 (3.33%) | |
| EGFR | 4/30 (13.33%) | |

**Supplementary Table S2:** Characteristics of participants with pulmonary metastases.

| **Primary origin of pulmonary metastases** | |
|---|---|
| Breast carcinoma | 8 / 29 (27.59%) |
| Colorectal carcinoma | 6/ 29 (20.69%) |
| Neuroendokrinne tumor and karcinoma | 3 / 29 (10.34) |
| Ovarian carcinoma | 1 / 29 (3.45%) |
| Pancreatic carcinoma | 1 / 29 (3.45%) |
| Melanoma | 2 / 29 (6.90%) |
| B non- Hodgkin lymphoma | 2 / 29 (6.90%) |
| T non- Hodgkin lymphoma | 1 / 29 (3.45%) |
| Renal carcinoma | 1 / 29 (3.45%) |
| Prostate carcinoma | 2 / 29 (6.90%) |
| Sarcoma | 1 / 29 (3.45%) |
| NUT midline carcinoma | 1 / 29 (3.45%) |

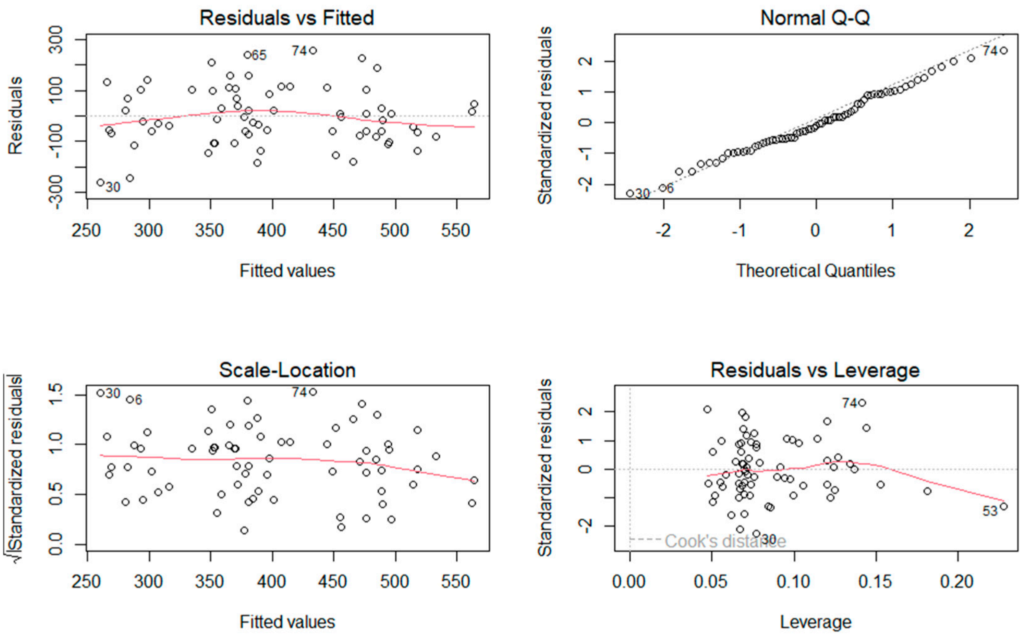


**Supplementary Figures S1-S4:** Diagnostic plots of the final linear regression model.

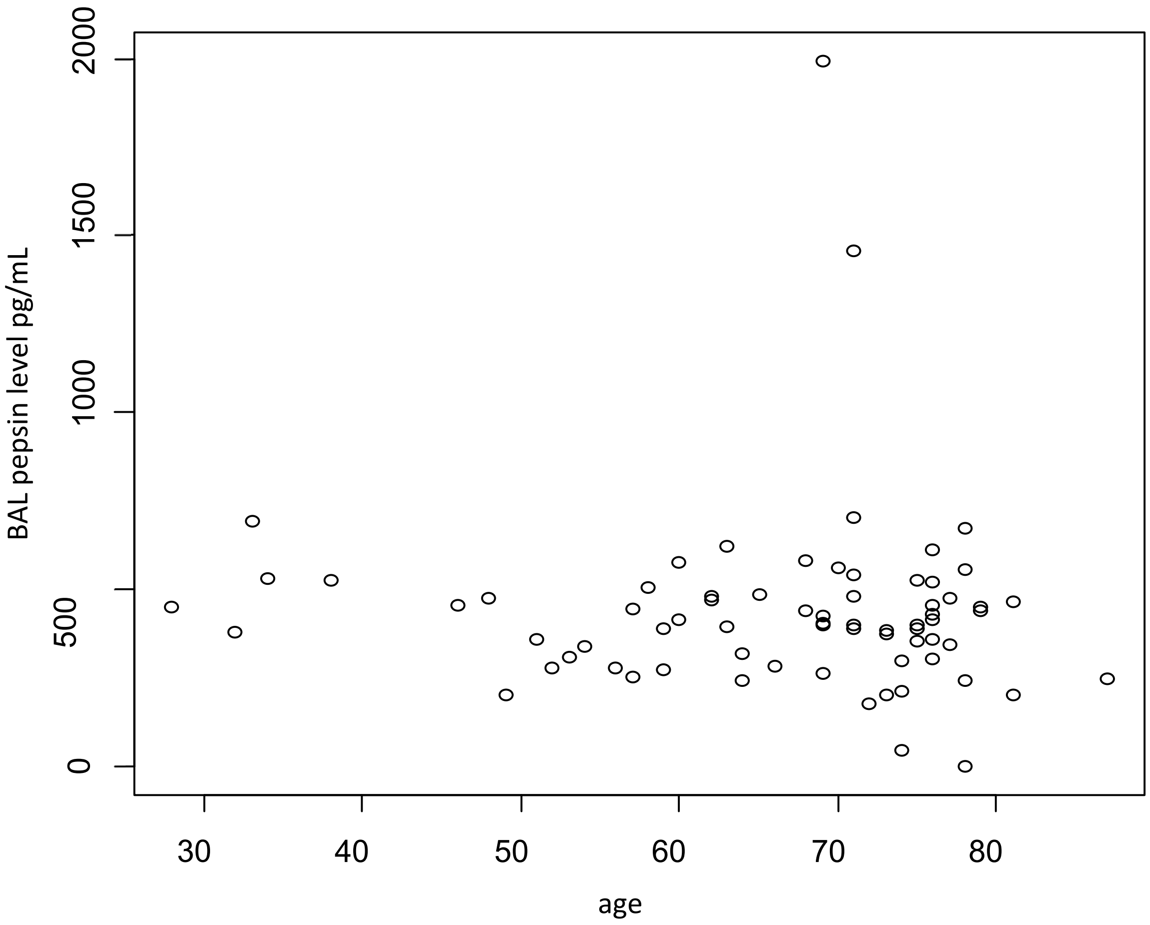


**Figure S5:** Correlation between BAL pepsin concentration and age.
